# Supplementary material for: Prevalence and Phenotypic Antimicrobial Resistance among ESKAPE Bacteria and Enterobacterales Strains in Wild Birds
Source: Antibiotics (Basel). 2022 Dec 15;11(12):1825. doi: 10.3390/antibiotics11121825 (PMC9774818; doi:10.3390/antibiotics11121825)
Supplement: Supplementary file 1 [file antibiotics-11-01825-s001.zip › Table S1 - Distribution and number-prevalence of isolates in bird species tested.pdf]

Table S1 - Distribution and number/prevalence of isolates in bird species tested

**Isolated strains** / **Bird species**	*E. faecium*	*S. aureus*	*P. aeruginosa*	*E. cloacae*	*E. aerogenes*	*K. pneumoniae*	*Salmonella* spp.	*E.coli*	*Citrobacter* spp.	*Proteus* spp.	*Serratia* spp.
Accipiter nisus		1		1				1			
Buteo buteo	2	1	3	2	1	3	1	11	2	1	1
Circaetus gallicus								1			
Anas platyrhynchos		1						2	2		
Anas clypeata											1
Cygnus olor								1			
Apus apus					1			1		1	1
Columba livia "domestica"	1			1			1	8	3		
Streptopelia decaocto					1			2	1		
Garrulus glandarius				1	1			1	1		
Pica pica				1				1			
Falco tinnunculus	2				1	1		9	3	2	1
Falco peregrinus	1							2			1
Carduelis carduelis	2	3	15		1			2	1		1
Coccothraustes coccothraustes								1	1		
Fulica atra								1			
Himantopus himantopus								1			
Scolopax rusticola					1			3	1		1
Athene noctua	1										
Bubo bubo								1			
Sylvia melanocephala									1		
Turdus merula								3	2		
Turdus philomelos								1			
Tyto alba								1			2
Upupa epops								1			
**Total strains (number)**	9	6	18	6	7	4	2	55	18	4	9
**Prevalence (%)**	5.5	3.6	11.0	3.6	4.2	2.4	1.2	33.7	11.0	2.4	5.5
**(95% CI %)**	2.71 - 10.5	1.50 - 8.19	6.85 - 17.1	1.50 - 8.19	1.81- 8.99	0.78 - 6.56	0.21 - 4.82	26.6 - 41.6	6.85 - 17.1	0.78 - 6.56	2.71- 10.5
