# Supplementary material for: Prevalence and Phenotypic Antimicrobial Resistance among ESKAPE Bacteria and Enterobacterales Strains in Wild Birds
Source: Antibiotics (Basel). 2022 Dec 15;11(12):1825. doi: 10.3390/antibiotics11121825 (PMC9774818; doi:10.3390/antibiotics11121825)
Supplement: Supplementary file 1 [file antibiotics-11-01825-s001.zip › Table S2 ¿C Results of antibiograms.pdf]

Table S2. Results of antibiograms enterobacterales strains

**Bird species**	Bacteria species	PRL	AMP	AMC	IMI	CRO	CAZ	CPD	AZM	C	CN	AK	TE	CIP	SXT	ESBL
Himantopus himantopus	*E. coli*	S	S	S	S	S	S	S	S	S	S	S	S	S	S	-
Turdus merula	*E. coli*	S	S	S	R	S	S	R	S	S	S	S	S	S	S	-
Turdus merula	*C. freundii*	S	R	R	S	S	S	S	S	S	S	S	S	S	S	-
Turdus merula	*E. coli*	S	S	S	S	S	S	S	S	S	S	S	R	S	S	-
Turdus merula	*C. freundii*	S	R	R	S	S	S	S	S	S	S	S	S	S	S	-
Turdus merula	*E. coli*	I	S	S	S	S	S	I	S	S	S	S	S	S	S	-
Buteo buteo	*E. coli**	R	R	R	S	R	R	R	R	S	I	S	R	R	R	-
Buteo buteo	*P. mirabilis**	S	R	S	I	R	S	S	R	S	S	S	R	S	S	-
Buteo buteo	*E. coli**	R	R	R	S	S	I	R	I	S	R	S	R	R	R	+
Buteo buteo	*S. enterica subsp. enterica*	I	S	S	I	R	R	R	S	S	S	S	S	I	S	-
Buteo buteo	*E. coli*	S	S	S	S	S	S	I	I	S	S	S	S	S	S	-
Buteo buteo	*E. coli**	R	R	R	S	S	R	R	S	I	S	S	R	S	R	+
Buteo buteo	*E. coli*	S	S	S	S	S	S	S	S	S	S	S	S	S	S	-
Buteo buteo	*E. coli**	I	R	R	S	R	S	S	S	S	S	S	S	S	S	-
Buteo buteo	*E. aerogenes*	S	R	R	S	S	S	S	S	S	S	S	S	S	S	-
Buteo buteo	*K. pneumoniae **	R	R	R	S	R	R	R	R	S	S	S	R	I	R	+
Buteo buteo	*K. pneumoniae **	R	R	R	S	R	S	R	R	S	S	I	R	I	R	+
Buteo buteo	*E. cloacae*	S	R	R	S	S	S	S	S	S	S	S	S	S	S	-
Buteo buteo	*E. coli*	S	S	S	S	S	S	S	S	S	S	S	S	S	S	-
Buteo buteo	*E. coli*	S	S	S	S	S	S	S	S	S	S	S	S	S	S	-
Buteo buteo	*E. cloacae*	S	R	R	S	S	S	S	S	S	S	S	S	S	S	-
Buteo buteo	*C. freundii*	R	R	R	S	S	R	R	S	S	R	R	R	R	R	+
Buteo buteo	*E. coli**	R	S	S	S	S	R	R	S	S	R	R	R	R	R	+
Buteo buteo	*K. pneumoniae **	R	R	R	S	R	R	R	S	S	S	R	R	R	R	+
Buteo buteo	*C. freundii**	R	R	R	S	R	R	R	S	S	I	R	R	R	R	-
Buteo buteo	*S. marcescens*	S	R	R	S	S	S	S	S	S	S	S	S	S	S	-
Buteo buteo	*E. coli**	R	S	R	S	R	R	R	S	S	S	R	R	R	R	-
Buteo buteo	*E. coli*	S	S	S	S	S	S	S	S	S	S	S	S	S	S	-
Falco tinnunculus	*C. freundii**	R	R	R	S	R	R	R	R	R	S	S	R	S	R	-
Falco tinnunculus	*K. pneumoniae **	R	R	S	S	R	I	R	R	S	S	S	R	S	R	+
Falco tinnunculus	*E. coli**	R	R	S	S	R	I	R	R	R	S	S	R	R	R	-
Falco tinnunculus	*E. aerogenes**	R	R	R	S	S	R	R	I	S	S	S	R	S	R	+

Falco tinnunculus	*E. coli**	I	R	S	I	I	S	S	I	S	S	S	R	S	R	-
Falco tinnunculus	*C. freundii**	I	R	R	R	I	S	I	R	S	S	S	R	S	R	-
Falco tinnunculus	*E. coli**	R	R	S	S	S	S	S	S	S	S	S	R	R	R	-
Falco tinnunculus	*P. vulgaris*	S	R	R	S	S	S	I	S	S	S	S	S	S	S	-
Falco tinnunculus	*E. coli*	R	R	S	S	S	S	S	S	S	S	R	S	S	I	-
Falco tinnunculus	*S. marcescens*	S	R	R	S	S	S	S	S	S	S	S	S	S	S	-
Falco tinnunculus	*E. coli*	S	S	S	S	S	S	S	S	S	S	S	S	S	S	-
Falco tinnunculus	*C. koseri*	S	R	S	S	S	S	S	S	S	S	S	S	S	S	-
Falco tinnunculus	*E. coli*	I	S	S	I	R	S	I	R	S	S	S	S	S	S	-
Falco tinnunculus	*E. coli*	S	S	S	S	S	S	S	S	S	S	S	S	S	S	-
Falco tinnunculus	*E. coli*	S	S	S	S	R	S	I	S	S	S	S	S	S	S	-
Falco tinnunculus	*E. coli**	R	R	S	S	S	S	S	S	S	S	S	R	R	R	-
Falco tinnunculus	*P. vulgaris*	S	R	S	S	S	S	S	S	S	S	S	S	S	S	-
Columba livia	*E. cloacae*	S	R	R	S	S	S	S	S	S	S	S	S	S	S	-
Columba livia	*E. coli*	R	S	S	S	S	S	I	S	S	S	S	S	S	S	-
Columba livia	*E. coli*	S	S	S	S	S	S	S	S	S	S	S	S	S	S	-
Columba livia	*S. enterica subsp. arizonae*	I	S	S	S	S	S	S	S	S	S	S	S	S	S	-
Columba livia	*C. freundii*	S	R	R	S	S	S	S	S	S	S	S	S	S	S	-
Columba livia	*E. coli*	S	S	S	S	S	S	S	S	S	S	S	S	S	S	-
Columba livia	*E. coli*	S	S	S	S	S	S	S	S	S	S	S	S	S	S	-
Columba livia	*E. coli*	S	S	S	S	S	S	S	S	S	S	S	S	S	S	-
Columba livia	*E. coli**	S	R	S	S	S	S	S	S	S	R	S	R	S	R	-
Columba livia	*E. coli*	R	S	S	S	S	S	I	S	S	S	S	S	S	S	-
Columba livia	*C. braakii*	S	R	R	S	S	S	S	S	S	S	S	S	S	S	-
Columba livia	*E. coli*	R	S	S	S	S	S	I	S	S	S	S	S	S	S	-
Columba livia	*C. freundii*	S	R	R	S	S	S	S	S	S	S	S	S	S	S	-
Apus apus	*P. vulgaris*	S	R	S	S	S	S	S	S	S	S	S	R	S	S	-
Apus apus	*E. aerogenes*	S	R	R	S	S	S	S	S	S	S	S	S	S	S	-
Apus apus	*E. coli*	S	S	S	I	S	S	S	S	S	S	S	R	S	S	-
Apus apus	*S. liquefaciens*	S	S	S	S	S	S	S	S	S	S	S	S	S	S	-
Streptopelia decaocto	*E. coli*	S	S	S	S	S	S	S	S	S	S	S	S	S	S	-
Streptopelia decaocto	*E. aerogenes*	S	R	R	S	S	S	S	S	S	S	S	S	S	S	-
Streptopelia decaocto	*E. coli*	S	S	S	S	S	S	S	S	S	S	S	S	S	S	-
Streptopelia decaocto	*C. braakii*	S	R	S	S	R	S	I	S	S	S	S	S	I	S	-

Fulica atra	*E. coli**	S	R	S	S	S	R	S	R	S	S	S	S	S	S	-
Bubo bubo	*E. coli*	I	S	S	S	I	S	I	I	S	S	S	S	S	S	-
Upupa epops	*E. coli*	S	S	R	S	S	S	S	S	S	S	S	S	S	S	-
Garrulus glandarius	*C. koseri*	S	R	S	S	S	S	S	S	S	S	S	S	S	S	-
Garrulus glandarius	*E. cloacae*	S	R	R	S	S	S	S	S	S	S	S	S	S	S	-
Garrulus glandarius	*E. coli*	S	R	S	S	S	S	S	S	S	S	S	S	S	S	-
Garrulus glandarius	*E. aerogenes*	S	R	R	S	S	S	S	S	S	S	S	S	S	S	-
Pica pica	*E. cloacae*	S	R	R	S	S	S	S	S	S	S	S	S	S	S	-
Pica pica	*E. coli*	S	S	S	S	S	S	I	S	S	S	S	S	S	S	-
Accipiter nisus	*E. cloacae*	S	R	R	S	S	S	S	S	S	S	S	s	S	S	-
Accipiter nisus	*E. coli*	S	R	S	S	S	S	S	I	S	S	S	S	S	S	-
Scolopax rusticola	*E. aerogenes*	S	R	R	S	S	S	S	I	S	S	S	S	S	S	-
Scolopax rusticola	*E. coli*	R	R	R	I	R	R	R	R	R	R	R	R	R	R	-
Scolopax rusticola	*E. coli*	R	R	R	I	R	R	R	S	S	S	S	S	S	R	-
Scolopax rusticola	*E. coli*	S	S	S	S	S	S	S	S	S	S	S	S	S	S	-
Scolopax rusticola	*S. marcescens*	I	R	R	S	S	S	S	S	S	S	S	R	I	S	-
Scolopax rusticola	*C. freundii*	S	R	R	S	S	S	S	S	S	S	S	S	S	S	-
Carduelis carduelis	*E. aerogenes*	I	R	R	S	S	S	S	I	S	S	S	S	S	S	-
Carduelis carduelis	*E. coli**	R	R	S	I	R	R	R	R	S	S	S	R	S	S	+
Carduelis carduelis	*S. liquefaciens*	R	S	S	S	R	I	I	I	S	S	S	S	S	S	-
Carduelis carduelis	*E. coli*	I	R	S	S	R	I	R	S	S	S	S	S	S	S	-
Carduelis carduelis	*C. braakii*	S	S	S	S	S	S	S	S	S	S	S	S	S	S	-
Coccothraustes coccothraustes	*C. koseri*	I	R	S	I	S	S	I	S	S	I	S	S	S	S	-
Coccothraustes coccothraustes	*E. coli*	S	S	S	S	S	S	I	S	S	S	S	S	S	S	-
Anas platyrhynchos	*E. coli*	S	R	S	S	S	S	S	S	S	S	S	S	S	S	-
Anas platyrhynchos	*C. koseri*	S	R	S	S	S	S	S	S	S	S	S	S	S	S	-
Anas platyrhynchos	*E. coli*	S	S	S	S	S	S	S	S	S	S	S	S	S	S	-
Anas platyrhynchos	*C. braakii*	S	S	S	S	S	S	S	S	S	S	S	S	S	S	-
Cygnus olor	*E. coli*	S	S	S	S	S	S	S	S	S	S	S	S	S	S	-
Anas clypeata	*S. odorifera*	S	S	S	S	S	S	S	S	S	S	S	S	S	S	-
Falco peregrinus	*E. coli**	I	R	S	S	S	I	S	R	S	I	S	R	S	R	-
Falco peregrinus	*S. odorifera*	R	R	S	S	S	S	S	R	S	S	S	S	S	S	-
Falco peregrinus	*E. coli**	R	R	R	R	R	R	R	R	S	R	S	R	R	R	-

Sylvia melanocephala	*C. freundii*	R	R	R	S	R	S	I	S	S	S	S	S	S	S	-
Circaetus gallicus	*E. coli*	S	S	S	S	S	S	S	S	S	S	S	S	S	S	-
Turdus philomelos	*E. coli*	I	S	S	I	R	R	R	S	S	S	S	S	I	S	-
Tyto alba	*E. coli*	S	S	S	S	S	S	S	S	S	S	S	S	S	S	-
Tyto alba	*S. odorifera*	S	S	S	S	S	S	S	S	S	S	S	S	S	S	-
Tyto alba	*S. odorifera*	S	R	S	S	S	S	S	S	S	S	S	S	S	S	-
	** multidrugs strains*	PRL	AMP	AMC	IMI	CRO	CAZ	CPD	AZM	C	CN	AK	TE	CIP	SXT	ESBL

PRL = piperacillin 100 µg, AMP = ampicillin, 10 µg; AMC = amoxycillin/clavulanic acid 30 µg; IMI= Imipenem 10 µg; CRO= ceftriaxone 30 µg; CAZ = ceftazidime, 30 µg; CPD= Cefpodoxime 10 µg; AZM= azithromycin 15 µg; C = chloramphenicol, 30 µg; CN = gentamicin, 10 µg; AK= amikacin 30 µg; TE = tetracycline, 30 µg; CIP = ciprofloxacin, 5 µg; SXT = sulphamethoxazole–trimethoprim, 1.25 + 23.75 µg; ESBL = Extended Spectrum Beta-Lactamase production

Table S3. Results of antibiograms *S. aureus* strains

Bird species	FOX	CN	E	TE	DO	CIP	DA	SXT
Accipiter nisus	R	S	S	S	S	S	R	S
Anas platyrhynchos	R	S	R	R	R	S	R	R
Buteo buteo	R	S	S	S	S	S	R	S
Carduelis carduelis	R	S	R	R	R	S	R	R
Carduelis carduelis	R	S	R	R	R	S	R	R
Carduelis carduelis	R	S	R	R	R	S	R	R

FOX= cefoxitin 30 µg; CN = gentamicin, 10 µg; E= erythromicin 15 µg; TE = tetracycline, 30 µg; DO = doxycycline, 30 µg; CIP = ciprofloxacin, 5 µg; DA= clindamycin 2 µg; SXT = sulphamethoxazole–trimethoprim, 1.25 + 23.75 µg;

Table S4. Results of antibiograms *Enterococcus* spp. strains

Bird species	P	E	DO	TE	CIP	RD	C
Athene noctua	I	S	S	S	S	I	S
Buteo buteo	R	R	R	R	S	R	S
Buteo buteo	R	R	R	R	S	R	S
Carduelis carduelis	R	S	S	S	S	I	S
Carduelis carduelis	S	S	S	S	S	S	S
Columba livia "domestica"	I	S	S	S	S	S	S
Falco tinnunculus	R	R	R	R	S	R	S
Falco tinnunculus	R	R	R	R	S	R	S
Falco peregrinus	I	S	S	S	S	S	S

P= penicillin 10 units; E= erythromicin 15 µg; DO = doxycycline, 30 µg; TE = tetracycline, 30 µg; CIP = ciprofloxacin, 5 µg; RD= rifampin 5 µg; C = chloramphenicol, 30 µg.

Table S5. Results of antibiograms *P. aeruginosa* strains

**Bird species**	PRL	CAZ	IMP	CT	CN	AK	CIP
Buteo buteo	S	S	S	S	S	S	S
Buteo buteo	S	S	S	S	S	S	S
Buteo buteo	S	S	S	S	S	S	S
Carduelis carduelis	S	S	S	S	S	S	S
Carduelis carduelis	I	S	S	S	S	S	S
Carduelis carduelis	I	S	S	S	S	S	S
Carduelis carduelis	S	S	S	S	S	S	S
Carduelis carduelis	S	S	S	S	S	S	S
Carduelis carduelis	S	S	S	S	S	S	S
Carduelis carduelis	I	S	S	S	S	S	S
Carduelis carduelis	I	S	S	S	S	S	S
Carduelis carduelis	S	S	S	S	S	S	S
Carduelis carduelis	S	S	S	S	S	S	S
Carduelis carduelis	I	S	S	S	S	S	S
Carduelis carduelis	I	S	S	S	S	S	S
Carduelis carduelis	S	S	S	S	S	S	S
Carduelis carduelis	S	S	S	S	S	S	S
Carduelis carduelis	S	S	S	S	S	S	S

PRL = piperacillin 100 µg; CAZ = ceftazidime, 30 µg; IMP = Imipenem, 10 µg; CN = gentamicin, 10 µg; CIP = ciprofloxacin, 5 µg; CT = colistin sulphate, 10 µg;
