# Supplementary material for: Prevalence and Phenotypic Antimicrobial Resistance among ESKAPE Bacteria and Enterobacterales Strains in Wild Birds
Source: Antibiotics (Basel). 2022 Dec 15;11(12):1825. doi: 10.3390/antibiotics11121825 (PMC9774818; doi:10.3390/antibiotics11121825)
Supplement: Supplementary file 1 [file antibiotics-11-01825-s001.zip › Table S3 - Classification of analysed wild birds species.pdf]

Table S3 – Classification of analysed wild birds species

Family	Common name	Scientific name	Number
Accipitridae	Eurasian Sparrowhawk	*Accipiter nisus*	2
	Common Buzzard	*Buteo buteo*	20
	Short-toed Snake Eagle	*Circaetus gallicus*	1
Anatidae	Mandarin Duck	*Aix galericulata*	1
	Mallard	*Anas platyrhynchos*	2
	Northern Shoveler	*Anas clypeata*	1
	Mute Swan	*Cygnus olor*	1
Apodidae	Common Swift	*Apus apus*	4
Ardeidae	Western Cattle Egret	*Bubulcus ibis*	1
Columbidae	Common Pigeon	*Columba livia "*domestica*"*	21
	Eurasian Collared Dove	*Streptopelia decaocto*	5
	Common Wood Pigeon	*Columba palumbus*	1
Corvidae	Eurasian Jay	*Garrulus glandarius*	2
	Eurasian Magpie	*Pica pica*	1
Falconidae	Common Kestrel	*Falco tinnunculus*	18
	Peregrine Falcon	*Falco peregrinus*	3
Fringillidae	European Goldfinch	*Carduelis carduelis*	51
	European Serin	*Serinus serinus*	1
	Hawfinch	*Coccothraustes coccothraustes*	1
Phasianidae	Common Pheasant	*Phasianus colchicus*	1
Rallidae	Eurasian Coot	*Fulica atra*	1
Recurvirostridae	Black-winged Stilt	*Himantopus himantopus*	1
Scolopacidae	Eurasian Woodcock	*Scolopax rusticola*	4
Strigidae	Little Owl	*Athene noctua*	2
	Eurasian Scops Ow	*Otus scops*	2
	European Eagle Owl	*Bubo bubo*	2
Sylviidae	Eurasian Blackcap	*Sylvia atricapilla*	1
	Sardinian Warbler	*Sylvia melanocephala*	1
Turdidae	Common Blackbird	*Turdus merula*	5
	Song Thrush	*Turdus philomelos*	1
Tytonidae	Western Barn Owl	*Tyto alba*	4
Upupidae	Eurasian Hoopoe	*Upupa epops*	1
**Total**			**163**
